# RALY regulate the proliferation and expression of immune/inflammatory response genes via alternative splicing of FOS

**DOI:** 10.1038/s41435-022-00178-4

**Published:** 2022-08-08

**Authors:** Zhao Liang, Aliya Rehati, Erhati Husaiyin, Dong Chen, Zhang Jiyuan, Buzukela Abuduaini

**Affiliations:** 1 Department of General Surgery, The First Affiliated Hospital of Xinjiang Medical University, Xinjiang, Urumqi 830054 China; 2 Department of Gastroenternology, The First Affiliated Hospital of Xinjiang Medical University, 393 South Li Yu Shan Road, Urumqi, Xinjiang 830054 China; 3 ABLife BioBigData Institute, Wuhan, Hubei 430075 China; 4 Xin Jiang Medical University, Urumqi, Xinjiang 830054 China; 5 Department of Intensive Care Unit, The First Affiliated Hospital of Xinjiang Medical University, 393 South Li Yu Shan Road, Urumqi, Xinjiang 830054 China

**Keywords:** Gene expression, Immunology

## Abstract

RALY is a multifunctional RNA-binding protein involved in cancer metastasis, prognosis, and chemotherapy resistance in various cancers. However, the molecular mechanism of which is still unclear. We have established RALY overexpression cell lines and studied the effect of RALY on proliferation and apoptosis in HeLa cells. Then we used RNA-seq to analyze the transcriptomes data. Lastly, RT-qPCR experiments had performed to confirm the RNA-seq results. We found that the overexpression of RALY in HeLa cells inhibited proliferation. Moreover, the overexpression of RALY changed the gene expression profile, and the significant upregulation of genes involved immune/inflammatory response related biological process by NOD-like receptor signaling pathway cytokine-cytokine receptor interaction. The significant downregulation genes involved innate immune response by the Primary immunodeficiency pathway. Notably, IFIT1, IFIT2, IFTI3, IFI44, HERC4, and OASL expression had inhibited by the overexpression of RALY. Furthermore, RALY negatively regulates the expression of transcription factors FOS and FOSB. Notably, we found that 645 alternative splicing events had regulated by overexpression of RALY, which is highly enriched in transcription regulation, RNA splicing, and cell proliferation biological process by the metabolic pathway. We show that RALY regulates the expression of immune/inflammatory response-related genes via alternative splicing of FOS in HeLa cells. The novel role of RALY in regulating immune/inflammatory gene expression may explain its function in regulating chemotherapy resistance and provides novel insights into further exploring the molecular mechanism of RALY in regulating cancer immunity and chemo/immune therapies.

## Background

RALY, known as a member of the Heterogeneous nuclear ribonucleoproteins (hnRNP) family belonging to RNA binding proteins. It was previously identified as a gene relevant to the embryonic lethality of homozygous lethal yellow mice [1]. The gene encodes an autoantigen that cross-reacts with EBNA-1 of the Epstein Barr virus is highly homologous to RALY, suggesting a potential function of RALY in viral response [2]. Importantly, transcript of RALY is overexpressed in various cancer tissues, furthermore, overexpression of RALY involve poor outcome in ovarian, lung, bladder, brain, and breast cancers [3]. The similar correlation between RALY overexpression and poor survival in hepatocellular carcinoma has also been reported. Meanwhile, a previous study reported that decreased of RALY expression is relevant with poor survival in clear cell renal cell carcinoma [4]. Another study showed that decreasing the expression of RALY inhibits cell proliferation and against aggressive biological behavior in hepatocellular carcinoma cells (HCC) [5]. Oxaliplatin is a third-generation platinum analog that kills cells by forming adducts on DNA, which inhibits the cancer cell replication and transcription [6]. Interestingly, down-regulation of RALY expression sensitizes cell lines of colorectal cancer treated with oxaliplatin without effect on the rate of cell growth, indicating the chemotherapeutic role of RALY [7]. These studies together support a general functional role of RALY in cancer development and progression.

Mechanistically, RALY was initially identified in spliceosomal complexes, indicating that RNA splicing could be involved [8]. RALY, together with the hnRNPs including hnRNPH/F, were also found to interact with RBFOX1/2 [9–11]. RALY, along with NONO/p54nrb16, was identified as an interactor of YB-1, an RNA-binding protein involving translational regulation of specific mRNAs [7]. These evidences strongly indicated the role of RALY in alternative splicing regulation. A proteomic study of the RALY-interacted proteins further suggested its role in regulation of mRNA metabolism and translational control [12]. Several recent studies indicate that RALY may bind poly-U stretches in vitro and in vivo [13, 14], which may associate with the differential expression of the target genes at the mRNA and protein levels [15]. RALY localization in the cytoplasmic compartment is found to co-sediment with ribosomes and polysomes, which may be related to its regulated mRNA and protein levels [16]. In fact, RALY primarily localizes in the nucleus and regulating transcription of many genes which involved in cell cycle and transcription regulation [16], as well as the alternative splicing of the pre-mRNA of PRMT1 and the metastasis of breast cancer cells [17]. RALY has been shown to be a direct target of miR-193a-3p, and a long non-coding RNA zinc finger antisense 1 may exert its oncogenic role as an sponge of miR-193a-3p and thusly releasing RALY for its action [18]. Nevertheless, the amplitude of RALY regulation of alternative splicing as an hnRNP protein and gene expression directly or indirectly has not yet been characterized.

This study set up a RALY overexpression HeLa cell model and obtained the RALY-regulated transcriptome from the control and RALY overexpression cells by an Illumina sequencing approach. We then analyzed RALY-regulated alternative splicing and gene expression in HeLa cells at the whole-transcriptome level. The results revealed 910 RALY-regulated AS events in genes that were strongly enriched in transcription regulation and predicted a mechanism of RALY to deregulate the cancer cell transcriptome. The expression levels of 910 genes were significantly changed in the RALY overexpression cell line, consistent with an indirect transcriptional regulation instead of mRNA stability change. It is noteworthy that RALY selectively increased the expression of genes functioning in virus response and inflammatory and innate immune response and repressed the expression of FOS and FOSB. Our results support a crucial and general role of RALY in regulating alternative splicing and gene expression, which explains some oncogenic and chemotherapeutic functions and predicts its role in defending against viral infection and regulating the immune response.

## Methods

### Cloning and plasmid construction

We designed primer pairs used for Hot Fusion by the CE Design (V1.04). For each of the primer, unique genomic sequences of RALY and the pIRES-hrGFP-1a vector sequence (17–30 nt) were included. The following sequences were the primer.

F-primer: TTCTGTGCACAAGGGCTATG

R-primer: ATGGCAGATGCTGCTCTCTT

EcoRI and XhoI (NEB) were used to digest the pIRES-hrGFP-1a vector at 37 °C for 2–3 h. The digested vector was obtained via 1.0% agarose gel and then purified by Qiagen column kit. And we isolated total RNAs by using Trizol, then purified RNA was reverse transcribed to cDNA by oligo-dT primer. And then the insert fragment was synthesized and amplified by PCR. Finally, we added the linearized vector constructed by *EcoRI* and *XhoI* (NEB) and the insert fragment to a PCR microtube for ligation with Clon Express® II One Step Cloning Kit (Vazyme). The constructed plasmid was transfected into *E. coli* by chemical transformation. The cloned RALY sequence was verified by Sanger sequencing.

### Cell culture and plasmid transfection

The HeLa cells were cultured with 5% CO_2_ at 37 °C in Dulbecco’s Modified Eagle’s Medium, in which containing 10% fetal bovine serum (Hyclone), penicillin (100 U/ mL), and streptomycin (100 g/mL). We then transfected the constructed RALY-overexpressed plasmid to HeLa cells by using Lipofectamine 2000 (Invitrogen, Carls-bad, CA, USA). The transfected HeLa cells were harvested after 48 h. The relative expression level of RALY was calculated and normalized to GAPDH mRNA level using 2^− ΔΔCT^ method [19].

### Cell proliferation and apoptosis experiments

We then performed MTT assay and Annexin-V/PI to assess the cell proliferation rate regulated by RALY overexpression.

### Western blot experiment

Western blot was also used to assess changes in protein levels. RALY overexpression and negative control HeLa cells were lysed in Ripa buffer. The sample was centrifuged, the supernatant was further treated on 10% sds-page gel, and then transferred to PVDF membrane (micropore). A monoclonal Flag antibody (SIG-MA-ALDRICH) was diluted in TBST (1:2000) and the RALY protein level was detected with Abclonal (1:2000) as load control.

### RNA-seq experiment

For RNA-seq library construction, total RNA was extracted from HeLa cells with TRIZOL (Ambion). Then removed the DNA by RQ1 DNase (Promega, Madison, WI, USA) and purified the RNA with two phenol-chloroform treatments. For each sample, the VAHTS Stranded mRNA-seq Library Prep Kit (Vazyme) was used to prepare library with 1 μg of the total RNA as input. Only polyadenylated RNAs were purified and fragmented for library. We then used Illumina HiSeq X Ten platform to obtain the sequences with 150 nt paired-end fastq format.

### RNA-seq filtering and alignment

After being obtained the raw reads, we removed the reads containing more than 2-N bases. Then adapter sequences and low-quality bases were trimmed from the remaining reads using FASTX-Toolkit (Version 0.0.13), quality filtered reads were aligned to the human GRCH38 genome by TopHat2 [20] allowing no more than 4 mismatches. Uniquely mapped reads were used for gene reads number counting and FPKM calculation [21].

### Differentially expressed genes (DEG) analysis

The R Bioconductor package edge R [22] was utilized to screen out the differentially expressed genes (DEGs). A false discovery rate <0.05 and fold change >2 or <0.5 were set as the cut-off criteria to screen out DEGs.

### Alternative splicing analysis

The alternative splicing events (ASEs) and regulated alternative splicing events (RASEs) between RALY overexpression and control samples were defined and quantified using the ABL as pipeline as described antecedently [23, 24]. Based on the splice junction reads, ten types of ASEs were detected, including ES, A5SS, A3SS, IR, MXE, 5pMXE, 3pMXE, cassette exon, A3SS&ES, and A5SS&ES.

### Reverse transcription qPCR validation of DEGs and AS events

To confirm the validity of the RNA-seq results, qRT-PCR was performed for some of the randomly selected DEGs. We used GAPDH as the internal control normalized the RNA expression levels of all the genes. Meanwhile, qRT-PCR assay was performed for ASE validation. We designed specific primers to detect alternative splicing events which were detailly described in ABL as method. The information of primers is presented in Additional file 1.

### Functional enrichment analysis

To explore the functions of DEGs and RASGs, using KOBAS 2.0 server [25] to identify the enriched Gene Ontology (GO) terms and KEGG pathways.

## Results

### RALY overexpression inhibits cell proliferation and which correlated with prognosis in cancer

**Fig. 1:**
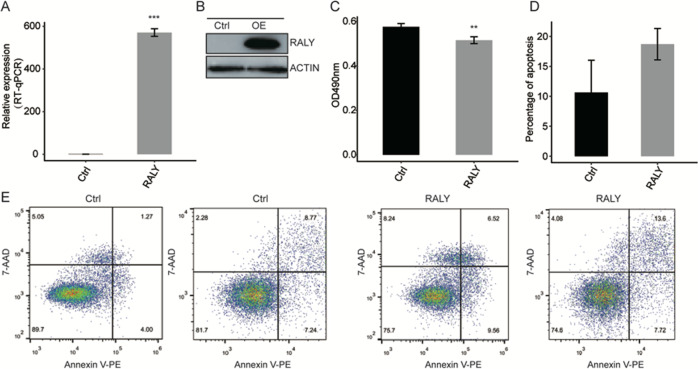
Overexpression of RALY impact on proliferation and apoptosis of HeLa cells. **A** RALY expression quantified by qRT-PCR. Error bars represent mean ± SEM. ***p < 0.001. **B** RALY was overexpressed and validated by Western blotting. **C** Overexpression of RALY suppressed the proliferation of HeLa cells. *P < 0.05, ***P < 0.001. **D** Overexpression of RALY promoted the cell apoptosis.

**Fig. 2:**
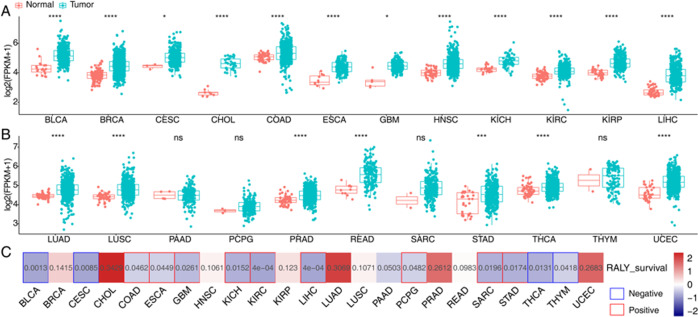
Analysis of RALY expression and survival in a number of cancers. **A** Levels of RALY expression were dramatically decreased in cancer tissues compared with corresponding normal tissues. **B** RALY expression is related with prognosis in cancer patients.

To explore the function of RALY at the cell level, we construct a functional cell model. RALY had overexpressed in the HeLa cell line. And overexpression of RALY was assessed through the qPCR and Western blot, which revealed that the expression level of RALY had upregulated in HeLa cells (Fig. 1A, B). The result of proliferation analysis showed that RALY overexpression inhibits cellular proliferation (Fig. 1C) and promoted the cell apoptosis (Fig. 1D, E). Analysis of RALY expression and survival in a number of cancers showed that the levels of RALY expression were dramatically decreased in cancer tissues compared with corresponding normal tissues (Fig. 2A, B) RALY expression is related with prognosis in cancer patients (Fig. 2C). These data suggest that overexpression of RALY involving the cellular proliferation, the mechanism of RALY in the cellular process still unclear, needs further investigation.

### RALY overexpression changes the genes expression profiles of HeLa cells

**Fig. 3:**
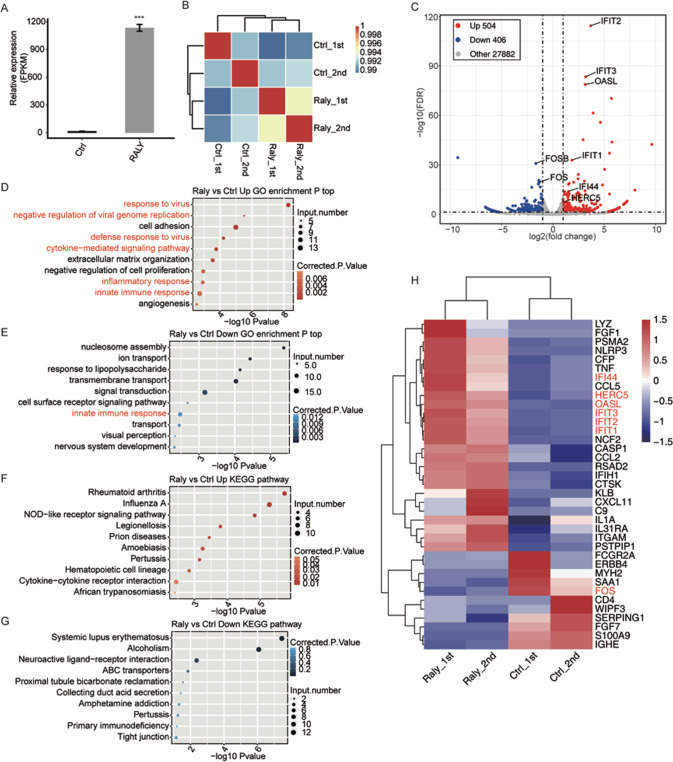
Analysis of the change in gene expression in response to RALY-OE. **A** RALY expression quantified by RNA-seq data. FPKM values were calculated as that has been explained in Materials and Methods. **B** Heat map shows the hierarchically clustered Person’s correlation matrix resulting from comparing the transcript expression values for control and RALY-OE values for control and RALY-OE samples. **C** Identification of RALY-regulated genes. Up-regulated genes are labeled in red, whereas down-regulated are labeled in blue in the volcano plot. **D** The top 10 representative GO biological processes of upregulated genes. **E** The top 10 representative GO biological processes of down-regulated genes. **F** The top 10 representative KEGG pathways of up-regulated genes. **G** The top 10 representative KEGG pathways of up-regulated genes. **H** Hierarchical clustering of DEGs in control and RALY overexpression samples. FPKM values are log2-transformed and then median-centered by each gene. Error bars represent mean ± SEM. ***p < 0.001.

To further explore the role of RALY on genes expressional and transcriptional, we constructed a total of four cDNA libraries prepared from control and RALY-OE cells (two biological replicated). The effective overexpression of RALY had confirmed with RNA‐seq data, which aligned with the RT‐qPCR results, FPKM values had used to generate a Pearson’s distance correlation matrix to contrast the transcriptomes from each sample. The FPKM values of RALY expression significantly upregulated in HeLa cells (Fig. 3A, B). The heatmap analysis of the DEG demonstrated that RALY-OE and the control were not significant distinguished (Fig. 3B). Based on the high-quality RNA-seq data from the RALY overexpression cells and control, the 504 upregulated and 406 downregulated significant differential expression genes were identified in RALY-OE compared to control (Fig. 3C). These results showed that RALY upregulation significantly changed the gene transcription.

### RALY overexpression selectively upregulates the transcription of viral response, inflammatory/innate immune response genes in HeLa cells

**Fig. 4:**
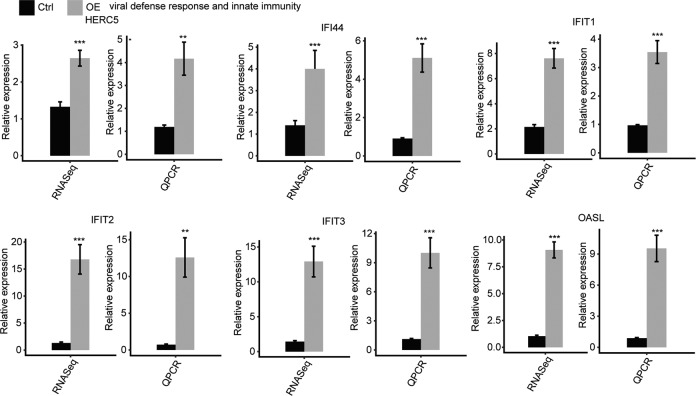
RALY regulates expression of genes associated with the viral defense response and innate immunity. Gene expression quantified by RNA sequencing data and qRT-PCR. Error bars represent mean ± SEM. ***p < 0.001.

To comprehensively explore the potential roles of 910 DEGs, we performed the GO and KEGG enrichment analysis. In total, 504 upregulated and 406 downregulated DEGs had identified. And the upregulated genes are mainly enriched in “cytokine-mediated signaling pathway,” “negative regulation of cell proliferation,” “inflammatory response,” “innate immune response” in GO enrichment (Fig. 3D). The KEGG analysis showed that the upregulated genes enriched in the “NOD-like receptor signaling pathway,” “cytokine-cytokine receptor interaction” (Fig. 3F). In contrast, the down-regulated genes enriched in the “innate immune response” in GO enrichment (Fig. 3E). And the KEGG pathways were most enriched in those associated with “primary immunodeficiency” (Fig. 3G). The heatmap analysis of the DEG demonstrated that RALY-OE and the control were not significant distinguished (Fig. 3H). The RNA-seq had performed to quantify the changes in mRNA levels in RALY-OE and Control, and we found that the six upregulated genes, including IFIT1, IFIT2, IFTI3, IFI44 HERC4, and OASL, were significantly upregulated in the RALY overexpression cell line (Fig. 4). These data indicated that RALY overexpression changed the transcription of proliferation and immune/inflammatory response-related genes.

### RALY overexpression downregulates the transcription of FOS and FOSB

**Fig. 5:**
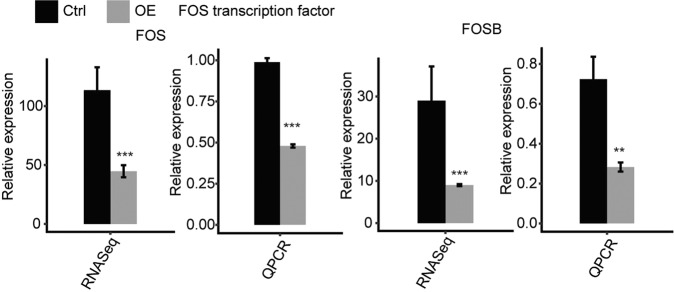
RALY regulates expression of FOS transcription factor. Gene expression quantified by RNA sequencing data and qRT-PCR. Error bars represent mean ± SEM. ***p < 0.001.

We performed qPCR had quantified the expression transcription factors (TFs) of FOS and FOSB in RALY-OE and control cells (Fig. 5). These two TFs were significantly suppressed under the RALY overexpression condition, demonstrating that RALY negatively regulates the expression of FOS transcription factors in HeLa cells. FOS will affect the immune/inflammatory response via changing the protein binding activity and ubiquitin-specific protease activity [26]. FOSB is 70% homology with Fos, which together with the Jun family members form the group of AP-1 proteins which modulated the gene expression in response to cytokines, growth factors, bacterial and viral infections [27]. Our results showed that RALY regulates immune/inflammatory response-related genes via the impact the transcription factor of FOS and FOSB expression.

### RALY overexpression selectively change the alternative splicing of transcription factor in HeLa cells

In order to deeply investigate the molecular function of RALY overexpression in HeLa cells, we analyzed the regulated AS events by RALY. The splicing reads from RALY overexpression and control HeLa cells were mapped to the reference genome, and 367,321 annotated exons (60.72% of total annotated ones) were observed. And a total of 162,055 known and 182,549 novel splice junctions, Meanwhile 20,446 known ASEs and 58,427 novel ASEs were detected.

**Fig. 6:**
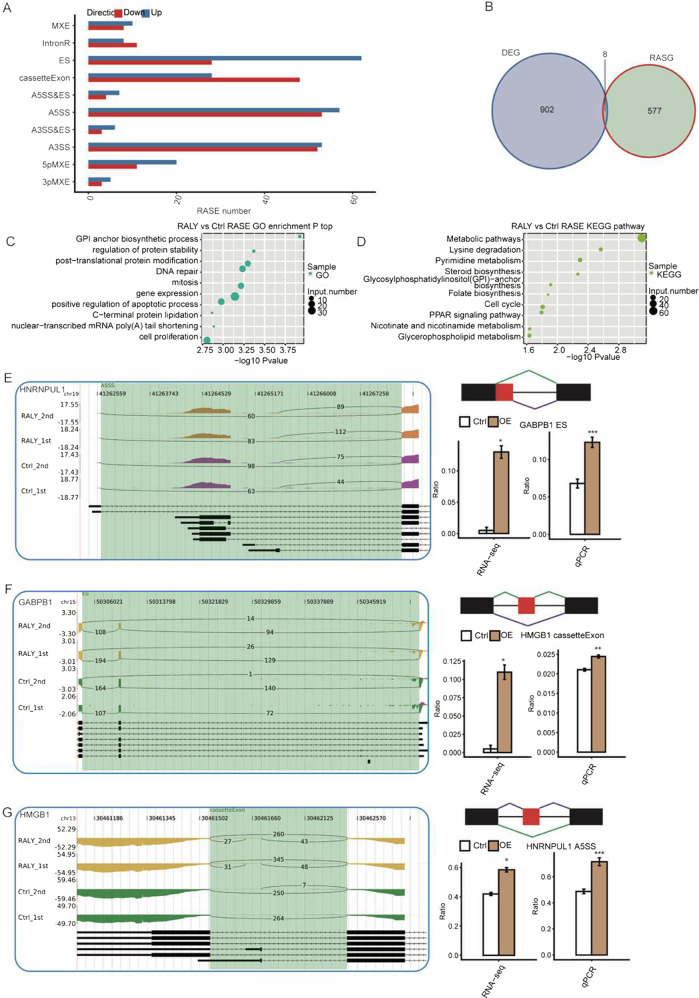
Validation of RALY regulated splicing events. **A** classification of different AS types regulated by the RALY. **B** overlap analysis between RALY regulated genes and splicing genes. **C** The Top 10 GO biological process analysis. **D** The Top 10 KEGG functional pathway of splicing gene. **E** ASEs in HNRNPUL. **F** ASEs in GABPB1. **G** ASEs in HMGB1. The IGV-sashimi plots showing the alternative splicing changes that occurred in the control or RALY overexpression HeLa cells. The results for ZNF638, HNRNPUL1, GABPB1, HMGB1, CTNND1, E4F1, ZSCAN32, SPOCD1, VEZF1, and ETV1 are presented. RNA-seq quantification and RT-PCR validation of alternative splicing regulation are shown at the bottom. *P < 0.05, **P < 0.01.

To further validate the high-confidence RALY-regulated alternative splicing events, AS ratio change between RALY overexpression and control cells contrasted by a custom pipeline, a total of 645 RASEs were identified, including 187 IR and 485 NIR RASEs. Among these RASEs, the number of A3SS, A5SS, IntronR, and ES was comparatively high (Fig. 6A). In the aggregate, 637 genes attributed to these RASEs had been identified as RALY-regulated alternative splicing genes after mapping and counting. These results showed that RALY regulated the alternative splicing events in HeLa cells. Eight genes were overlapping between DEGs and RASEs (Fig. 6B). And identified eight such genes: CCDC17, RP11-610P16.1, RP11-91A18.4, MYH16, CTD-2587H24.5, IFIT1, LAMC2, PAK6. The GO biological process analysis showed that the overlapped gene mainly enriched in response to the virus, defense response to the virus, cytokine-mediated signaling pathway, negative regulation of protein binding, regulation of transcription, DNA-dependent, apoptotic process, mitotic cell cycle. Enriched KEGG pathway included those involved in Focal adhesion, T cell receptor signaling pathway, Axon guidance. These data indicated that upregulation of RALY expression level had affected the alternative splicing of DEGs annotated with the immune/inflammatory response.

To investigate the function of RASGs, we performed GO analysis, and there were 203 RASGs enriched in protein binding and 67 RASGs with DNA binding. Furthermore, the RASGs enhanced in GO biological process, including transcription DNA dependent, regulation of transcription from RNA polymerase II promoter, regulation of RNA splicing, cell proliferation, gene expression, regulation of translational (Fig. 6C). The enriched pathways include the peroxisome, Nucleotide excision repair, Metabolic pathways (Fig. 6D). To further investigate the function of RALY overexpression on alternative splicing events, 8 RASEs had analyzed by qPCR, and the changes in the ratio of 8 RASEs in qPCR were consistent with the RNA-Seq results (Fig. 6E). These RASEs occurred in ZNF638, HNRNPUL1, GABPB1, HMGB1, CTNND1, E4F1, ZSCAN32, SPOCD1, VEZF1, and ETV1.

## Discussion

Alternative splicing is a ubiquitous regulatory mechanism of gene expression; in precursor mRNA, various splice sites had selected to generate functionally distinct mRNA and protein variants [28], which widely expand metazoan genomes’ functional and regulatory ability [15]. The dysregulation of cancer-associated splice variants may play a critical role in cancer cellular behavior, including cellular proliferation and cellular death it is a potential target for therapeutic intervention [29]. As reported, alternative splicing mediated cervical cancer oncogenesis, indicating the novel therapeutic target for the treatment of cervical cancer [30]. We found that the overexpression of RALY inhibited cell proliferation and impacted the expression of immune-inflammatory response-related genes at the transcript level, and involved regulating transcription factors activity. These data indicated that RALY promoted the immune response gene expression through transcriptional regulatory mechanism correlated with RNA binding activity.

RALY has upregulated various types of tumors. Its downregulation reduces cell proliferation [16]. However, another study reported that the increased level of RALY promoted proliferation, migration, and invasion abilities in HCC cells [15]. These results indicated that RALY is a tumorigenesis oncogene in mammalian cells. Notably, our data also revealed that RALY overexpression inhibited cell proliferation in HeLa cells, inconsistent with previous reports. And the transcriptome analysis revealed that RALY changed the expression of proliferation and immune/inflammatory-related genes. Furthermore, the FOS and FOSB, which belong to the Fos family of AP-1 transcription factors, inhabited RALY-OE cell lines. As reported, the FOS and FOSL1 were involved in regulating genes governing progression by the cell cycle and, consequently, are both associated with promoting proliferation [31, 32]. As reported, FOSL is a critical mediator in cancer cell EMT/MET plasticity. These results together indicated that RALY involved the proliferation biological process via regulation of the alternative splicing and transcriptor factors of FOS in HeLa cells.

In this study, we found that RALY promoted the expression of IFIT1, IFIT2, IFTI3, IFI44, HERC4, and OASL. The previous research reported that IFIT1, IFIT2, IFTI3, IFIT3, IFI44, HERC4, and OASL genes are antitumoral effects and modulated the inflammatory response in cancer and infection [5, 18]. OASL can boost innate host defense and improve immunity [33]. IFIT1 and IFITM3 expression were related to several immune checkpoint molecules and tumor-associated macrophage markers [34]. As reported, IFIT1 regulated different stages of the host innate resistant response duration of both viral and bacterial infection and may modulate the inflammatory response in human astrocytes [35]. IFI44 is a type I interferon-inducible gene family [36], participating in microtubule formation, involving an autoimmune response and inhibiting proliferation [37]. These results indicated that overexpression of RALY impacted the expression of inflammatory/immune response and proliferation-related genes in HeLa cells. The immune system has been defined as a crucial component of the immune surveillance of cancer and plays an essential role in cancer progression and tumorigenesis [38]. RALY overexpression inhibited the immune/inflammatory-related genes expression at transcription level might be associated with the chemo treatment resistance in cancer cell biological process.

In addition, Systemic inflammatory response may accelerate the progression of cancer and distant metastasis by a diversity of mechanisms. Based on the above evidence, we may speculate that RALY is involved in cancer immunity. This founding widened our current knowledge of the critical role of RALY in regulating the immune/ inflammatory response in cancer. As reported, RALY is modulated in both alternative splicing and translation [39]. In this study, transcriptome analysis suggested differential expression in genes related to the immune-inflammatory response. Meanwhile, we have detected RALY-dependent alternative splicing of transcription factors and splice factors. ZNF638 is a transcriptional coactivator that operates as an early regulator of adipogenesis in vitro [40]. HNRNPUL 1 and 2 are recruited to sites of DNA damage in an RNA-independent manner and promote adequate DNA resection [41]. GABPB1 is a transcription factor subunit. The higher expression levels of GABPB1 had associated with poor prognostic in renal cancer [42]. HMGB1 is a nuclear protein that acts as an intriguing molecule in inflammatory disorders and promotes inflammation through elucidated signal and molecular transport mechanisms [43]. CTNND1 overexpression in HCC cells induced EMT, invasion, and migration traits in vitro and boosted metastatic capacity in vivo [44]. E4F1 was a central metabolic node that regulated pyruvate oxidation and promoted the tricarboxylic acid cycle to meet energy demand [45]. SPOCD1 encodes a protein that pertains to the transcription factor S-II (TFIIS) family of transcription factors and accelerates the proliferation and metastasis of glioma cells by up-regulating PTX3 [46]. VEZF1 has expressed in the anterior-most mesoderm, and Its expression is later restricted in the vascular endothelium [47]. In addition, Ets-transcription factor ETV1 is a viral oncogene, and stromal expansion in PDAC contributed to the development of larger primary tumors. Furthermore, the KEGG analysis showed several tumorigeneses and immune-response relevant signaling pathways. Notably, most genes in this pathway are involved in the immune-inflammatory response. These results highlight that RALY regulates the inflammatory response through a metabolic pathway with alternative splicing. However, RALY selectively regulates the alternative splicing of transcription factors mainly associated with immune/inflammatory response and tumorigenesis in HeLa cells, which affect the cellular process, including proliferation and apoptosis, and metabolism in cancer cells.

## Conclusion

In summary, we concluded that RALY overexpression inhibits proliferation in HeLa cells. Simultaneously, RALY regulates the expression of immunity and inflammatory response genes by modulating the splicing of regulators factors and alternative splicing, which might correlate with tumorigenesis in HeLa cells. Which data provides evidence for further investigation of the function of RALY in cancer immunology and clinical resistance in cancer advance under the given therapy. Nevertheless, there are some limitations to our study. Further studies are required to verify the molecular function of RALY in cancer metabolism. Further investigation of the role of all RALY proteins in carcinogenesis, especially in vivo and in vitro, will be more significant and preferred the new perspectives in cancer treatment.

## Availability of data and material

All data generated or analyzed during this study have been included in this published article and its supplementary information files. The datasets supporting the results of this article are available in the NCBI Gene Expression Omnibus and are accessible through GEO series accession number (GSE157125).

## Supplementary information

Additional file 1

Additional file 2

Additional file 3

Additional file 4

Additional file 5

Additional file 6

Additional file 7

Additional file 8

Additional file 9

Additional file 10

Additional file 11

Additional file 12

supplement legend

Additional file 13
