# Supplementary material for: RALY regulate the proliferation and expression of immune/inflammatory response genes via alternative splicing of FOS
Source: Genes Immun. 2022 Aug 8;23(8):246–54. doi: 10.1038/s41435-022-00178-4 (PMC9758052; doi:10.1038/s41435-022-00178-4)
Supplement: Supplementary file 13 — supplement legend [file 41435_2022_178_MOESM13_ESM.docx]

Additional files

Additional file1:Additional file 1(gRT-PCR primers ).xIsx

Additional file2:DEG_overlap.xls

Additional file3:Down_GO_annotation_P.xIs

Additional file4:Down GO enrichment P.xls

Additional file5:Down_KEGG_pathway_iden.xls

Additional file6:expressed gene_FPKM.txt

Additional file7:known_AS_ IR.txt

Additional file8:known_AS_ NIR.txt

Additional file9:Obtain_the_high_quality_clean_reads.xls

Additional file10:Raly_vS_Ctrl_IR_RAS_p0.05.txt

Additional file11:Raly_vs_Ctrl_NIR_RAS_p0.05.txt

Additional file12:Raly_vs_Ctrl_Sig_DEG.txt

Additional file13:Up KEGG pathway iden.xls
